# Neural control of anticipatory braking and lateral balance predicts fall risk in older adults: BMI-dependent mechanisms

**DOI:** 10.3389/fnagi.2025.1699584

**Published:** 2026-01-12

**Authors:** Youjung Choi, Jiyoung Jeong, Hyungji Lee, Hyang Jun Lee, Ji Won Han, Ki Woong Kim

**Affiliations:** 1 Department of Brain and Cognitive Science, Seoul National University College of Natural Science, Seoul, Republic of Korea; 2 Department of Neuropsychiatry, Seoul National University Bundang Hospital, Sungnam, Republic of Korea; 3 Department of Innovative Science and Engineering, Underwood International College, Yonsei University, Incheon, Republic of Korea; 4 Department of Psychiatry, Seoul National University College of Medicine, Seoul, Republic of Korea; 5 Institute of Human Behavioral Medicine, Seoul National University Medical Research Center, Seoul, Republic of Korea

**Keywords:** body composition, gait biomechanics, neural control, preventative health care, prospective cohort

## Abstract

**Introduction:**

Falls are a leading cause of disability among older adults, and neural control is increasingly recognized as a critical risk factor. In this prospective cohort study, we investigated whether deceleration during terminal swing (DCEL), and medial-lateral velocity zero crossing count (VZCC), reflecting anticipatory braking and lateral balance control, predict future falls, and whether body mass index (BMI) moderates these associations. A total of 380 older adults were assessed and stratified by BMI (overweight: ≥ 25 kg/m^2^).

**Methods:**

Fallers (*n* = 68) and non-fallers (*n* = 312) were identified prospectively over a 24-month follow-up period. Gait was measured using inertial measurement units (IMUs) during 14-meter walks.

**Results:**

Compared to non-fallers, fallers were older (75.1 ± 4.31 vs. 73.4 ± 4.51 years, *p* = 0.002), had more prior falls, more negative DCEL values (−10.57 ± 15.61 vs. -5.08 ± 16.39 cm/s, *p* = 0.010), and higher VZCC (4.96 ± 2.59 vs. 4.29 ± 1.31, *p* = 0.023). In multivariate models, age, prior falls, DCEL, VZCC, and the BMI × DCEL interaction predicted fall risk. DCEL predicted falls only in individuals with normal weight (OR = 0.533, 95% CI = 0.368–0.770, *p* = 0.001), whereas VZCC predicted falls across all BMI levels. Gait parameters reflecting neural control predict fall risk, with effects moderated by BMI.

**Discussion:**

Anticipatory braking control is critical for individuals with normal weight, whereas lateral instability elevates fall risk regardless of BMI. These findings highlight the value of BMI-stratified fall assessments and targeted interventions.

## Introduction

Falls are a critical public health challenge in aging populations. Approximately one-third of community-dwelling adults aged 65 or older experience at least one fall annually. Beyond physical injuries, falls often lead to functional decline, institutionalization, and mortality (Montero-Odasso et al., 2022; Beck Jepsen et al., 2022). Understanding the complex mechanisms that contribute to fall risk is essential for developing targeted interventions.

Emerging evidence has challenged the traditional musculoskeletal-centric view of fall prevention, suggesting that safe ambulation depends not only on motor processes but also on higher-order cognitive processes. There is increasing recognition of the link between cognitive decline and reduced mobility, with executive dysfunction emerging as a stronger predictor of falls than conventional gait metrics (Mirelman et al., 2012).

Gait control in older adults relies on complex cortical–subcortical networks that integrate executive control and attention functions with motor commands via the basal ganglia and brainstem (Takakusaki, 2017). These pathways are vulnerable to age-related neurodegeneration such as ischemia and inflammation (Parihar et al., 2013). Recent advances in wearable sensor technologies have enabled more precise and ecologically valid assessment of gait, providing proxy measures of neural control capacity (Byun et al., 2016). These sensors allow for the detection of early, subclinical changes in motor control and objective monitoring of gait in real-world environments. This enhances sensitivity to neural dysfunction compared to traditional parameters (Byun et al., 2023).

Traditional spatiotemporal gait parameters have shown inconsistent associations with fall risk. They primarily reflect musculoskeletal function rather than underlying neural control mechanisms. For instance, step width variability has sometimes shown inconsistent associations with fall histories, failing to distinguish between adaptive compensatory strategies and pathological instability (Brach et al., 2005). Similarly, step length variability is inconsistently predictive and does not surpass traditional assessments of muscle strength or balance in forecasting falls (Silva et al., 2024). To address these limitations, we focused on two sensor-derived gait parameters that are sensitive to neural control processes rather than just their spatiotemporal outcomes.

First, deceleration during the terminal swing phase (DCEL), defined prior to heel strike anticipatory braking control. Although pendulum-like gait dynamics assist forward progression (Bauby and Kuo, 2000), active anticipatory control during terminal swing is required to position the limb for safe heel strike. When this control deteriorates, the body undergoes an abrupt stop only at the moment the swing foot contacts the ground, instead of performing proper deceleration (Chastan et al., 2009). This parameter specifically captures the active neural control process occurring during anticipatory motor planning, distinguishing it fundamentally from traditional spatiotemporal parameters. Older fallers often have shown such impaired deceleration, failing to successfully recover balance (Pijnappels et al., 2005). Anticipatory motor planning critical for DCEL relies heavily on prefrontal-striatal circuits, which coordinate the timing and execution of planned movements through feedforward control mechanisms (Vink et al., 2005).

Second, the frequency of zero crossings in medial-lateral (ML) COM velocity reflects feedback-driven neuromuscular mechanisms mediated by the vestibular and proprioceptive systems (Wang et al., 2024). Traditional ML measures emphasize the magnitude of sway rather than dynamics of neural corrective processes for lateral balance (Hausdorff, 2005), whereas VZCC captures the temporal dynamics of corrective responses by quantifying the frequency of directional changes in COM velocity. Similarly, zero crossing analysis based on center of pressure signals in the ML direction effectively detects impaired postural control and degraded neuromuscular regulation (Quijoux et al., 2021; Picoli et al., 2022). Feedback-driven lateral balance control measured by VZCC is primarily governed by the cerebellum and brainstem vestibular nuclei, which process sensory feedback and generate rapid corrective responses (MacKinnon, 2018). While measures like maximum Lyapunov exponent are powerful indicators of gait stability, they require extensive computational resources (Reynard and Terrier, 2014). VZCC offers a simpler yet sensitive alternative for quantifying corrective dynamics and is more feasible for clinical implementation while maintaining sensitivity to neural control deficits. These distinct neural pathways suggest that DCEL and VZCC may capture complementary aspects of fall risk – anticipatory versus reactive control mechanisms.

While neural control mechanisms are crucial to fall risk, body mass index (BMI) may fundamentally influence the interpretation of neural control gait parameters. Excess body mass increases trunk momentum and neuromuscular demands, potentially interfering with the precision of balance control (Dinkel et al., 2019; Ferrucci et al., 2002). Thus, BMI may not only directly contribute to fall risk but also fundamentally affect how gait parameters reflect underlying neural control mechanisms.

The relationship between neural control-related gait parameters and fall risk is likely not linear across different BMI levels. Individuals with higher BMI demonstrate adaptive neuromuscular responses due to the biomechanical modifications associated with mobilizing greater body mass (Maktouf et al., 2024). Conversely, individuals with lower BMI, particularly with sarcopenia may have compromised neuromuscular capacity (Yeung et al., 2019). These biomechanical differences suggest that optimal cut-off values or risk stratification based on DCEL and VZCC should be BMI-specific rather than applying universal standards.

Despite increased interest in the interplay between gait control, neural regulation, and BMI, few studies have examined whether BMI moderates the relationship between neural control-related gait parameters and fall risk. A better understanding of these moderation effects could inform the development of personalized fall prevention strategies and assessment tools that account for the complex interplay between neural control capacity and BMI related biomechanical constraints.

Based on the neural control framework outlined above, we hypothesized that: (1) more negative DCEL values would be associated with increased fall risk, as insufficient anticipatory deceleration reflects deteriorated motor planning capacity compromising safe heel strike; (2) higher VZCC values would predict increased fall risk, reflecting greater frequency of motion corrections; (3) BMI would moderate these effects, with the predictive strength of DCEL and VZCC varying across BMI levels.

## Materials and methods

### Participants

We analyzed baseline gait data from 380 community-dwelling, non-demented older Koreans aged 55 years or older (197 men and 183 women) who participated in a prospective gait study conducted among participants enrolled in two population-based longitudinal cohort studies: the Korean Longitudinal Study on Cognitive Aging and Dementia (KLOSCAD) (Han et al., 2018) and the Korean Frailty and Aging Cohort Study (KFACS) (Lee et al., 2022) (213 participants from KLOSCAD and 167 participants from KFACS). A previous study using these combined cohorts has demonstrated comparable demographic and gait characteristics between KLOSCAD and KFACS participants, supporting the validity of pooled analyses (Lee et al., 2022). The study size was determined based on the number of eligible participants after applying defined exclusion criteria (Supplementary Figure S1). This study was conducted as a prospective dynamic cohort study with rolling enrollment. All gait assessments used in the present study were newly and prospectively collected through a separate IRB-approved gait study. Participants were consecutively recruited between July 2016 and November 2022. Upon enrollment, each participant underwent a baseline gait assessment. Incident falls were prospectively monitored via single question during telephone interviews every 6 months over a 24-month following each participant’s baseline gait assessment, so incident falls were unknown at the time of gait data collection. The prospective design and independent ascertainment of incident falls were intended to minimize recall and reverse-causation bias. Incident falls were defined as unexpected events in which a person comes to rest on the ground or floor, not due to a major intrinsic event or overwhelming external hazard (Tinetti et al., 1988). The incident falls were treated as the dependent outcome variable in all inferential analyses. History of falls within the 12 months prior to baseline gait assessment was evaluated separately and coded as a binary variable (yes/no). All participants had normal or corrected-to-normal vision and were free from major psychiatric, neurological, or musculoskeletal disorders that could affect gait or fall risk. We used the World Health Organization (WHO) classification for BMI (overweight BMI ≥ 25 kg/m^2^) to maintain comparability with international literature (World Health Organization, 2000). All participants provided written informed consent themselves or via their legal guardians. The study was approved by the Institutional Review Board of Seoul National University Bundang Hospital (IRB: B-1603/338-301) and conducted in accordance with the Declaration of Helsinki. This manuscript was prepared in accordance with the Strengthening the Reporting of Observational Studies in Epidemiology (STROBE) guidelines.

### Gait assessment

As described in our previous work (Lee et al., 2022), gait was measured using inertial measurement units (IMUs) (FITMETER^®^ [FitLifeInc., Suwon, Korea] or ActiGraph^®^ [SMD solution, Seoul, Korea]), which were 35 × 35 × 13 mm (14 g) or 30 × 40 × 10 mm (17 g) and the GAITRite (CIR Systems Inc., Havertown, PA) simultaneously. The IMUs were smooth edges with a hexahedron design. The IMUs contained a digital tri-axial accelerometer (BMA255, BOSCH, Germany) and gyroscope (BMX055, BOSCH, Germany). Three IMUs were attached to participants at the L3-L4 lumbar vertebrae (the approximate COM) and on the dorsum of both feet over the 2nd-3rd metatarsal region to identify heel strike timing and capture trunk motion. All sensors belonged to the same multi-sensor acquisition system and were hardware-synchronized. Each participant walked straight along a 14-meter walkway at a self-selected pace, turned around at the end, then walked back to the starting point. This procedure was repeated three times, and the average values across all valid trials were used for analysis. We placed the GAITRite electronic mat in the middle for the walkway to measure steady-state walking. The GAITRite is a USB-connected portable gait assessment platform equipped with an electronic walkway that records spatiotemporal gait parameters at 100 Hz.

### Data processing and analysis

Following validated methods for tri-axial accelerometry-based gait analysis in older adults, we calculated spatiotemporal gait parameters (Lee et al., 2022; Byun et al., 2019). Acceleration data were extracted using ActiGraph software (SMD solution, Seoul, Korea) and analyzed from CSV files using MATLAB (The MathWorks Inc., Natick, MA). Step length and step width obtained using a GAITRite pressure-sensitive walkway system, which directly measures footfall positions based on embedded sensors. These metrics were included to characterize biomechanical gait adaptations across BMI groups. We quantified DCEL as the difference between the velocity of the COM at the onset of terminal swing and at heel strike. $\text{DCEL}=V_{\text{terminl swing onset}}-V_{\text{heel strike}}$ . Velocity was derived by integrating vertical axis of trunk acceleration. Thus DCEL reflects vertical COM deceleration, and its interpretation as forward braking pertains to impact attenuation and momentum regulation rather than directional measurement. Heel strike was identified as the absolute minimum peak of vertical acceleration recorded by the foot sensor (Byun et al., 2019). The deceleration onset was defined as the point where vertical velocity began to decrease during terminal swing, occurring approximately 13% before heel strike (Guimarães et al., 2021). To minimize integration drift, acceleration data were detrended by subtracting the mean within each step. After numerical integration, mean velocity within each step was removed again to zero-center the profile, which reduces cumulative bias from sensor noise and offset. More negative DCEL values indicate insufficient reduction of vertical COM velocity prior to heel strike, reflecting impaired anticipatory braking control. A schematic of the DCEL computation procedure is provided in Supplementary Figure S2 to aid visual interpretation. For each identified time point, velocity was calculated by integrating the acceleration values of the COM, using the same drift-minimization procedure described above. Building upon previous work demonstrating that zero crossing features quantify motor irregularities (Quijoux et al., 2021; Picoli et al., 2022), we calculated the VZCC of the COM velocity as an indicator of ML balance control. VZCC was defined as the total number of zero crossings in the ML velocity signal of the COM, normalized by each participant’s step length. Higher VZCC values indicate greater ML instability and more frequent corrective movements. A schematic illustrating an example ML velocity trace used to derive VZCC is shown in Supplementary Figure S3. Raw IMU signals were preprocessed in MATLAB (The MathWorks Inc.) to extract gait metrics (DCEL, VZCC, timing events).

### Statistical analysis

Normality was examined using the Shapiro–Wilk test. Because several variables violated normality assumptions, Mann–Whitney U tests were used for continuous variables, while χ^2^ tests were used for categorical variables between fallers and non-fallers, as well as between normal weight (BMI < 25 kg/m^2^) and overweight (BMI ≥ 25 kg/m^2^) groups. To explore potential moderating effects of BMI on the relationship between gait variables and fall risk, interaction terms were included in logistic regression models.

The full model included sex, age, education, Mini-Mental State Examination (MMSE), history of falls, BMI, DCEL, VZCC, and interaction terms (BMI × DCEL, BMI × VZCC, DCEL × VZCC, and BMI × DCEL × VZCC) as independent variables. Age and sex were included as covariates rather than interaction terms to avoid overfitting given limited subgroup sample sizes. Interaction terms were tested only for BMI because moderation by BMI status was the primary hypothesis of interest. BMI was modeled as a continuous variable in the full-sample logistic regression to examine does-response associations and interaction effects. Because our moderation hypothesis concerned differences between normal weight and overweight individuals, BMI was dummy-coded (0 = normal weight BMI, 1 = overweight) when constructing interaction terms. Thus, the main effect of BMI was modeled as a continuous predictor, whereas BMI was dummy-coded only for the construction of interaction terms. For clinical interpretability, we additionally performed stratified analyses using BMI categories (normal vs. overweight), where BMI served solely as a grouping variable rather than a continuous covariate. The three-way interaction term (BMI × DCEL × VZCC) was included in the initial full model as an exploratory analysis to examine whether the combined effects of both gait parameters might vary by BMI. This exploratory approach allowed us to identify the most parsimonious model through systematic evaluation of interaction effects. Given the exploratory nature of these interaction analyses, results should be interpreted with caution.

To identify gait-related predictors of fall risk within each BMI group, stratified logistic regression analyses were performed. We calculated average marginal effects (AMEs) to quantify how the effects of DCEL and VZCC on fall risk varied across BMI levels. Additionally, Johnson-Neyman analysis was used to identify the BMI thresholds at which the effects of gait variables became significant. Analyses were conducted using complete cases and no imputations was performed for missing data. Loss to follow-up was minimal, and participants with incomplete follow-up data were excluded from inferential analyses. No formal sensitivity analyses were conducted. All analyses were performed using R version 4.5.0 (Foundation for Statistical Computing, Vienna, Austria) and the Statistical Package for the Social Sciences for Windows version 25 (IBM Corp., Armonk, NY). No inferential statistics were conducted in MATLAB; MATLAB was used for preprocessing and signal-level feature extraction. Statistical significance was set at *p* < 0.05.

## Results

### Participant characteristics

Among the 380 participants, 68 (17%) experienced at least one fall during the 24-month follow-up period. The overweight group (BMI ≥ 25 kg/m^2^) comprised 124 participants (32.6%). Table 1 presents participant characteristics stratified by fall status and BMI group. Compared to non-fallers, fallers were older (75.13 ± 4.31 vs. 73.37 ± 4.51 years, *p* = 0.002) and had a higher prevalence of previous falls (26.47% vs. 16.02%, *p* = 0.035). Women showed a marginally greater incidence of falls in unadjusted comparisons (*p* = 0.062), but this effect was not significant after covariate adjustment (*p* = 0.198). Importantly, fallers demonstrated significantly more negative DCEL values (−10.57 ± 15.61 vs. -5.08 ± 16.39 cm/s, *p* = 0.010) and higher VZCC values (4.96 ± 2.59 vs. 4.29 ± 1.31, *p* = 0.023), indicating impaired anticipatory braking control and greater ML instability, respectively. VZCC remained significant in fully adjusted models (*p* = 0.046), indicating that ML instability contributes to fall risk beyond demographic differences. The overweight group had significantly shorter step length (58.25 ± 7.72 vs. 60.10 ± 6.99 cm, *p* = 0.034), wider step width (9.53 ± 2.71 vs. 8.95 ± 2.53 cm, *p* = 0.042), and higher VZCC values (4.77 ± 2.19 vs. 4.24 ± 1.23, *p* = 0.017) compared to the normal weight group. DCEL values did not differ significantly between BMI groups (*p* = 0.065). A detailed flow of participant inclusion and exclusion is presented in Supplementary Figure S1.

**Table 1:** Baseline participant characteristics.

Variables	All	Incident fall^*^			BMI		
	(*N* = 380)	No (*N* = 312)	Yes (*N* = 68)	*p^†^*	Normal weight (*N* = 256)	Overweight (*N* = 124)	*p^†^*
Age (years, mean ± SD)	73.68 ± 4.56	73.37 ± 4.51	75.13 ± 4.31	**0.002**	73.58 ± 4.65	73.90 ± 4.38	0.449
Women, *n*	183 (48.20)	144 (46.15)	39 (57.35)	0.062	124 (48.43)	59 (47.58)	0.875
Men, *n*	197 (51.80)	168 (53.85)	29 (42.65)		132 (51.56)	65 (52.42)	
Education (years, mean ± SD)	13.60 ± 3.42	13.60 ± 3.36	13.62 ± 3.73	0.742	13.80 ± 3.34	13.19 ± 3.57	0.080
MMSE (points, mean ± SD)	27.41 ± 2.07	27.42 ± 1.95	27.34 ± 2.60	0.720	27.43 ± 2.00	27.35 ± 2.27	0.876
BMI (kg/m^2^, mean ± SD)	23.92 ± 2.32	23.97 ± 2.33	23.67 ± 2.29	0.299	22.64 ± 1.48	26.56 ± 1.27	**< 0.001**
History of falls^‡^, *n* (≥ yes/no per participant)	68 (17.90)	50 (16.02)	18 (26.47)	**0.035**	48 (18.75)	20 (16.13)	0.532
Step length (cm, mean ± SD)	59.50 ± 7.28	59.72 ± 7.04	58.48 ± 8.29	0.315	60.10 ± 6.99	58.25 ± 7.72	**0.034**
Normalized step length^#^ (cm/cm, mean ± SD)	0.36 ± 0.04	0.36 ± 0.03	0.36 ± 0.04	0.897	0.37 ± 0.03	0.36 ± 0.04	**0.008**
Step width (cm, mean ± SD)	9.14 ± 2.60	9.20 ± 2.64	8.86 ± 2.44	0.204	8.95 ± 2.53	9.53 ± 2.71	**0.042**
DCEL ^¶^ (cm/s, mean ± SD)	−6.06 ± 16.37	−5.08 ± 16.39	−10.57 ± 15.61	**0.010**	−6.95 ± 16.46	−4.22 ± 16.08	0.065
VZCC^§^ (counts, mean ± SD)	4.41 ± 1.63	4.29 ± 1.31	4.96 ± 2.59	**0.023**	4.24 ± 1.23	4.77 ± 2.19	**0.017**

Incident falls shown descriptively as follow-up outcomes. BMI, body mass index; SD, standard deviation; MMSE, Mini Mental State Examination; DCEL, deceleration of center of body mass velocity during terminal swing phase; VZCC, velocity zero crossing counts. Continuous variables are presented as (mean ± standard deviation) and categorical variables as number (percentage). ^*^Incident falls during the 24-month follow-up. ^†^Continuous variables were compared using Mann–Whitney U tests due to violation of normality assumptions, and categorical variables were compared using chi square test. ^‡^History of falls was defined as a binary variable indicating whether participants experienced ≥1 fall within 12 months prior to baseline assessment. ^#^Step length was additionally normalized to height. ^¶^The difference in vertical velocity of the center of body mass between the onset of the terminal swing and heel strike. ^§^The number of zero crossing in the medial-lateral velocity of the center of body mass normalized by step length. Bold values indicate statistical significance at the *p* < 0.05 level.

### Predictors of fall risk

**Figure 1:**
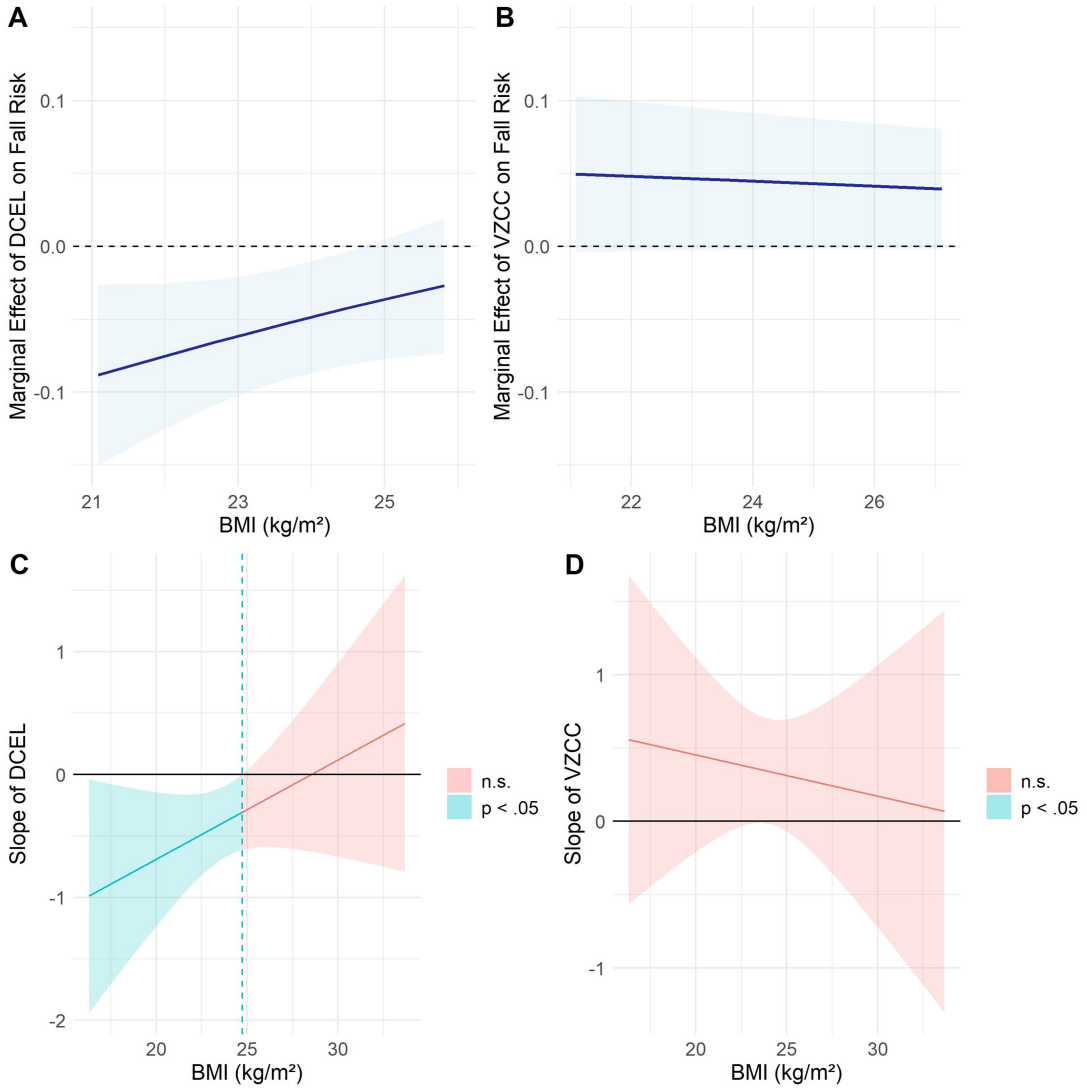
Average marginal effects and Johnson–Neyman significance regions for deceleration during terminal swing (DCEL) and medial–lateral velocity zero crossings (VZCC) across the body mass index (BMI) distribution. **(A)** Average marginal effects of DCEL on fall risk. **(B)** Average marginal effects of VZCC on fall risk. **(C)** Johnson–Neyman plot showing BMI thresholds at which the association between DCEL and fall risk becomes significant. **(D)** Johnson–Neyman plot showing BMI thresholds for the association between VZCC and fall risk. Fall risk was operationalized using prospectively monitored incident falls. Shaded regions represent 95% confidence intervals; horizontal dashed lines denote the null effect (*β* = 0). DCEL, deceleration in the center of body mass during terminal swing phase; VZCC, velocity zero-crossing counts; BMI, body mass index.

Table 2 shows the results of multivariate logistic regression analysis using incident falls as outcome. Several factors were significant predictors of fall risk: age (OR = 1.102, 95% CI = 1.031–1.178, *p* = 0.004), history of falls (OR = 2.073, 95% CI = 1.072–4.012, *p* = 0.030), DCEL (OR = 0.547, 95% CI = 0.383–0.781, *p* = 0.001), VZCC (OR = 1.595, 95% CI = 1.007–2.525, *p* = 0.046), and the BMI × DCEL interaction (OR = 2.062, 95% CI = 1.047–4.061, *p* = 0.036). Importantly, the significant BMI × DCEL interaction indicates that the relationship between DCEL and fall risk varies according to BMI levels. As shown in Figure 1A, the inverse association between DCEL and fall risk was stronger among individuals with lower BMI and weaker at higher BMI levels. This relationship became statistically non-significant beyond a BMI threshold of 24.71 kg/m^2^, which is close to the cutoff of 25 kg/m^2^ used to define normal weight in the current study. The Johnson-Neyman analysis confirmed this moderation effect (Figure 1C). In contrast, VZCC showed a consistent positive effect on fall risk across all BMI levels (Figure 1B) although the 95% confidence intervals for the BMI moderation effect included zero, indicating no statistically significant moderation (Figure 1D).

**Table 2:** Effects of gait parameters, BMI, and their interactions on prospective fall risk.

Variables	*β* ^*^	SE^*^	*p* ^*^	OR (95% CI)^*^
Constant	−9.019	4.094	0.028	< 0.001
Sex	0.434	0.337	0.198	1.544 (0.797–2.989)
Age	0.097	0.034	**0.004**	1.102 (1.031–1.178)
Education	0.040	0.047	0.397	1.041 (0.949–1.141)
MMSE	0.026	0.072	0.714	1.027 (0.891–1.183)
History of falls	0.729	0.337	**0.030**	2.073 (1.072–4.012)
BMI	−0.063	0.065	0.337	0.939 (0.826–1.068)
DCEL^†^	−0.604	0.182	**0.001**	0.547 (0.383–0.781)
VZCC^‡^	0.467	0.234	**0.046**	1.595 (1.007–2.525)
BMI×DCEL	0.724	0.346	**0.036**	2.062 (1.047–4.061)
BMI×VZCC	−0.122	0.299	0.684	0.885 (0.493–1.591)
DCEL×VZCC	0.093	0.254	0.714	1.098 (0.667–1.807)
BMI×DCEL×VZCC	−0.126	0.357	0.723	0.881 (0.438–1.772)

*β*, regression coefficient; SE, standard error; OR, odds ratio; CI, confidence interval; MMSE, Mini Mental State Examination; BMI, body mass index; DCEL, deceleration of center of body mass velocity during terminal swing phase; VZCC, velocity zero crossing counts. ^*^All variables were entered into a single binary logistic regression model. Coefficients represent estimates from a single multivariate model including all listed variables simultaneously. ^†^The difference in vertical velocity of the center of body mass between the onset of the terminal swing and heel strike. ^‡^The number of zero crossing in the medial-lateral velocity of the center of body mass normalized by step length. Bold values indicate statistical significance at the *p* < 0.05 level.

### Stratified analysis by BMI group

To further explore this moderating effect of BMI, stratified analyses were conducted in Table 3. In the normal weight group, both DCEL (OR = 0.533, 95% CI = 0.368–0.770, *p* = 0.001) and VZCC (OR = 1.702, 95% CI = 1.053–2.752, *p* = 0.030) were significant predictors of fall risk. Specifically, more negative DCEL values and higher VZCC values were associated with increased fall risk. In contrast, among the overweight group, neither DCEL (OR = 1.149, *p* = 0.645) nor VZCC (OR = 1.404, *p* = 0.103) significantly predicted falls. Notably, history of falls predicted future falls only in the normal weight group (OR = 2.827, *p* = 0.008), but was not significant in the overweight group (OR = 0.632, *p* = 0.574).

**Table 3:** Differential effects of gait parameters on fall risk in normal weight versus overweight older adults.

Variables	*β* ^*^	SE^*^	*p* ^*^	OR (95% CI)^*^
Normal weight (*N* = 256)				
Constant	−13.798	4.392	0.002	< 0.001
Sex	0.426	0.418	0.308	1.531 (0.674–3.478)
Age	0.113	0.040	**0.005**	1.120 (1.035–1.211)
Education	0.085	0.061	0.165	1.088 (0.966–1.226)
MMSE	0.081	0.088	0.361	1.084 (0.912–1.288)
History of falls	1.039	0.393	**0.008**	2.827 (1.308–6.114)
DCEL^†^	−0.630	0.188	**0.001**	0.533 (0.368–0.770)
VZCC^‡^	0.532	0.245	**0.030**	1.702 (1.053–2.752)
DCEL×VZCC	0.090	0.256	0.725	1.094 (0.662–1.808)
Overweight (*N* = 124)^¶^				
Constant	−3.780	6.563	0.565	0.023
Sex	0.345	0.609	0.571	1.412 (0.428–4.659)
Age	0.053	0.068	0.433	1.055 (0.923–1.205)
Education	−0.047	0.077	0.539	0.954 (0.821–1.109)
MMSE	−0.060	0.113	0.594	0.942 (0.754–1.175)
History of falls	−0.459	0.815	0.574	0.632 (0.128–3.125)
DCEL^†^	0.139	0.302	0.645	1.149 (0.636–2.076)
VZCC^‡^	0.339	0.208	0.103	1.404 (0.934–2.108)
DCEL×VZCC	−0.035	0.250	0.889	0.966 (0.592–1.575)

*β*, regression coefficient; SE, standard error; OR, odds ratio; CI, confidence interval; BMI, body mass index; MMSE, Mini Mental State Examination; DCEL, deceleration of center of body mass velocity during terminal swing phase; VZCC, velocity zero crossing counts. ^*^Binary logistic regression analysis adjusting for sex, age, education, MMSE score, and previous history of falls. ^†^The difference in vertical velocity of the center of body mass between the onset of the terminal swing and heel strike. ^‡^The number of zero crossing in the medial-lateral velocity of the center of body mass normalized by step length. ^¶^Overweight is defined as BMI over 25 kg/m^2^. Bold values indicate statistical significance at the *p* < 0.05 level.

## Discussion

This study provides novel insights into fall risk mechanisms in older adults by examining gait parameters reflecting anticipatory braking control (DCEL) and lateral balance control (VZCC). The goal of our study was to determine whether these parameters predict prospective fall risk and whether these associations are moderated by BMI. Using longitudinal cohort data with prospective 24-month fall follow-up, we successfully identified gait-related characteristics linked to future falls in community-dwelling older adults.

Our findings revealed that older adults who experienced incident falls after gait assessment exhibited more negative DCEL values and higher VZCC values, indicating impairments in both anticipatory braking control and ML stability. These results support our hypothesis that diminished neuromuscular control during gait contributes to fall risk. More negative DCEL values reflected deficient deceleration capacity during the terminal swing phase, where the body fails to properly slow down before heel strike, thereby increasing fall risk. This interpretation aligns with prior research emphasizing braking force as a critical gait parameter for stability (Chastan et al., 2009; Pijnappels et al., 2005).

Consistent with previous research (Ferrucci et al., 2002), the overweight group exhibited shorter step length, wider step width, and higher VZCC values compared to the normal weight group, while DCEL did not significantly differ by BMI group. In multivariate regression models, older age, history of falls, more negative DCEL values, and higher VZCC values independently predicted greater fall risk.

While these individual parameter effects are important, the most notable finding was the moderating effect of BMI on the relationship between DCEL and fall risk. Because BMI functions as a continuous physiological factor and as a categorical moderator, our analytic strategy incorporated both approaches. The significant BMI × DCEL interaction (OR = 2.062, 95% CI = 1.047–4.061, *p* = 0.036) indicated that the association between DCEL and fall risk diminished with increasing BMI. Johnson-Neyman analysis showed that DCEL was significantly associated with fall risk only among individuals with BMI below 24.71 kg/m^2^, suggesting that effective anticipatory braking control is particularly critical for those with the normal weight range.

This differential pattern may be interpreted through the lens of ‘motor reserve.’ Normal-weight individuals may rely more heavily on precise feedforward control (anticipatory braking). Consequently, they may be more sensitive to subtle declines in neural planning capacity captured by DCEL. In contrast, overweight individuals may benefit from the inertial stability provided by increased body mass, effectively dampening the immediate impact of minor deceleration errors, provided they can maintain their compensatory wide-based gait. (Browning, 2012; Stoffregen, 2016). This suggests that in normal-weight older adults, fall risk is primarily driven by neural control deficits, while in the overweight individuals, it is more influenced by the ability to manage biomechanical constraints.

When considering VZCC, a more nuanced pattern emerges. The stratified analyses confirm the moderating effect of BMI on the relationship between neural control-related gait parameters and fall risk, indicating that these gait markers are more predictive in individuals with normal weight than in those who are overweight. The pattern observed for VZCC (significant in the overall model and normal weight group but not in the overweight group) suggests a nuanced relationship. While VZCC did not show a significant BMI × VZCC interaction in the full model, the stratified analysis reveals that its predictive power may be attenuated in overweight individuals, possibly due to reduced statistical power or biomechanical compensation strategies. The Johnson-Neyman analysis (Figure 1D), showing confidence intervals that include zero across all BMI levels reflects this complex pattern where VZCC effects, while not formally moderated by BMI, may still vary in practical significance across the BMI spectrum.

To understand why BMI moderates the DCEL-fall risk relationship, several biomechanical mechanisms may explain these differential patterns. First, overweight individuals face increased trunk inertia during walking, which requires greater muscular effort for deceleration control (Browning and Kram, 2007). This mechanical demand may exceed their capacity for precise neural control of deceleration during terminal swing phase, potentially explaining why DCEL becomes a less reliable indicator of fall risk in the overweight population. Second, the overweight individuals often adopt compensatory gait strategies, including shorter step length and wider base of support. These adaptations serve as successful biomechanical compensations, reducing reliance on terminal swing deceleration to maintain stability (Lai et al., 2008). In contrast, normal weight individuals rely more on precise neuromuscular timing and coordination.

Unlike DCEL, VZCC showed consistent associations with fall risk across all BMI levels. Although the Johnson-Neyman analysis did not identify a significant BMI threshold for VZCC, regression results demonstrated that higher VZCC values consistently predicted elevated fall risk regardless of BMI range. This is consistent with prior studies showing that greater ML sway of the COM leads to increased gait variability and reduced dynamic stability in older adults and neurologically impaired populations (Engel et al., 2025; Quijoux et al., 2021; Picoli et al., 2022). The neurobiological significance of increased VZCC warrants deeper examination. The increased ‘zero crossings’ reflect a ‘busier’ or more ‘noisy’ control signal, potentially indicating instability in the feedback loops involving the cerebellum, vestibular system, and proprioceptive pathways that govern lateral stability. This increased frequency of corrective actions, even when the overall magnitude of sway is not large, signifies an inefficient and potentially fragile control system.

While increased ML acceleration is often attributed to biomechanical compensations, such as the ‘wide-based gait’ or ‘waddle’ observed in obesity and aging (Browning, 2012; McCrory et al., 2014; Stoffregen, 2016; Baumgart et al., 2016; Álvarez-Millán et al., 2023), VZCC captures a distinct dimension of stability: neural control efficiency. A wide-based gait primarily increases lateral displacement magnitude. However, if the movement is smooth and well-planned, it should not necessitate frequent directional changes. In contrast, an elevated VZCC indicates frequent, rapid corrective oscillations. Our findings show that overweight individuals exhibit both wider step width (mechanical compensation) and higher VZCC (neural inefficiency), suggesting that mechanical adaptations alone are insufficient to stabilize the center of mass without imposing a heavy burden on feedback-driven neural control loops.

Such inefficiency in lateral control represents a fundamental breakdown in the subcortical feedback mechanisms that maintain dynamic stability (Mancini and Horak, 2010). The cerebellum, which plays a crucial role in predictive control and motor adaptation, may struggle to generate appropriate corrective responses when bombarded with noisy sensory input (Takakusaki, 2017). Similarly, degraded vestibular function, common in aging, may lead to delayed or inappropriate corrective actions, manifesting as increased VZCC. This makes VZCC a sensitive marker for fall risk regardless of body mass, as it captures the quality of neural control rather than just biomechanical outcomes.

The sensitivity of VZCC to neural control mechanisms provides several advantages over traditional measures. Unlike conventional ML stability measures—such as root-mean-square sway, or local dynamic stability —which focus on magnitude, VZCC quantifies the temporal dynamics of corrective responses, making it more sensitive to early neuromuscular control changes (Mancini and Horak, 2010). Moreover, VZCC can be derived from shorter gait assessments with simpler computations compared to local dynamic stability, offering a practical clinical tool (Reynard and Terrier, 2014). These technical advantages are supported by neurobiological evidence.

Supporting evidence from signal processing studies and research on neurodegenerative disorders has shown that increased high-frequency components in COM sway have been associated with impaired motor coordination and fall risk (Engel et al., 2025; Horak and Mancini, 2013; Reimann et al., 2018). The sensitivity of VZCC to high-frequency components of ML sway patterns suggests its ability to capture subcortical, feedback-driven neural control mechanisms, supporting its integration into clinical fall risk models for detecting subtle neuromuscular impairments in aging populations (Kannan et al., 2023).

These mechanistic insights translate into specific clinical recommendations. Our findings support BMI-stratified intervention approaches that account for the primary drivers of fall risk in each population. For normal weight older adults, comprehensive gait analysis including DCEL and VZCC provides valuable insights into neural control deficits. In contrast, for individuals with higher BMI, assessments should focus on biomechanical markers and compensatory strategies. Beyond its direct effects, BMI emerges as a critical modifier of gait-fall risk relationships. Previous studies have reported a U-shaped relationship, where both underweight and obese individuals face elevated risk through distinct biomechanical mechanisms (Neri et al., 2020; Trevisan et al., 2019). Higher BMI is linked to increased trunk momentum during perturbations and reduced muscle strength relative to body weight, impairing balance recovery (Russell and Hamill, 2010; Gao et al., 2019), while underweight status may reflect sarcopenia and frailty, leading to reduced neuromuscular capacity for postural control (Yeung et al., 2019).

Contrary to expectations, history of falls predicted future falls only among normal-weight individuals, whereas the association was not significant in the overweight group. Notably, the lack of significant predictors in the overweight subgroup does not necessarily indicate absence of biomechanical or neural mechanisms. Rather, reduced subgroup sample size and low event rate likely hindered detection of statistical significance. Future studies with larger overweight and obsess cohorts are needed to determine whether these associations truly differ by BMI. This counterintuitive finding may represent a Type II error due to the smaller sample size in the overweight subgroup (*n* = 124, with only 20 reporting prior falls). The wide confidence interval (0.128–3.125) reflects substantial uncertainty in this estimate. Alternatively, this may reflect the behavioral modifications specific to the overweight individuals who, after experiencing falls, might adopt more cautious gait patterns or compensatory strategies, such as shorter step length or wider step width, potentially reducing future fall risk. In our sample, the overweight group demonstrated shorter step length and wider step width compared to the normal weight group, supporting the possibility of biomechanical compensatory mechanisms. Future studies with larger cohorts of overweight individuals are needed to definitively establish whether BMI truly moderates the relationship between fall history and future fall risk.

While these findings advance our understanding of BMI-gait-fall relationships, several limitations of our study should be noted. First, we used the WHO threshold of BMI ≥ 25 kg/m^2^ to define overweight. Although Asian population-specific cutoffs (BMI ≥ 23 kg/m^2^) are often considered more appropriate, our identified threshold of 24.71 kg/m^2^ falls within the WHO normal range. This consistency provides confidence in our findings. The consistency between our statistically derived threshold and the WHO classification supports the robustness of the BMI moderation effect. However, an important limitation is that the overweight group in our sample did not include individuals with severe obesity; only three participants had BMI ≥ 30 kg/m^2^, and none exceeded BMI 35 kg/m^2^. Thus, the moderation effect should be interpreted within this restricted range. Future studies including more participants with higher BMI and greater adiposity variability are needed to clarify whether the moderation effect persists across the full BMI spectrum. Similarly, although the sample spanned a broad age range, fall events within each age group were too few to support age-stratified moderation analyses. Thus, larger age-specific samples will be required to evaluate potential age-dependent effects. Additionally, because direct measures of body composition such as muscle mass, fat distribution were not collected, BMI served as the sole anthropometric indicator. This limits our ability to differentiate whether specific body composition components contribute uniquely to fall risk. Future research should consider factors such as muscle mass, strength, fat distribution, or bone density to provide more insights into biomechanical moderators of fall risk. Second, this study focused on community-dwelling older adults without major neuropsychological or musculoskeletal disorders that could affect gait or fall risk. The generalizability of our findings to clinical populations with specific conditions requires further investigation. Third, BMI, DCEL, and VZCC were measured only once at baseline; therefore repeated assessments are needed to determine how changes in these variables influence fall risk over time.

Building on these findings and limitations, future studies should integrate neural control-related gait metrics with neuroimaging and neurophysiological assessments in older adults. This multimodal approach would enable a more comprehensive understanding of the neural mechanisms contributing to fall risk and how changes in brain function impact gait and balance control. Ultimately, this approach may enable brain-based, personalized strategies for fall prevention and rehabilitation, tailored to the complex interplay between neural control capacity, biomechanical constraints, and body composition.

This study demonstrates that neural control-related gait parameters, DCEL and VZCC, predict fall risk in community-dwelling older adults, with BMI significantly moderating these relationships. Our findings reveal that anticipatory braking control (DCEL) is particularly predictive of falls among normal weight individuals, while lateral balance control (VZCC) maintains predictive value across BMI levels. From a clinical perspective, measures derived from wearable sensors offer more precise and ecologically valid assessments of motor control during walking—the context in which most falls occur (Byun et al., 2023; Alvarez et al., 2023). This approach enables detection of subtle neural control deficits missed by traditional assessment and offers insights into real-time movement control.

Our findings support BMI-stratified intervention approaches. For normal weight older adults, targeted interventions focusing on anticipatory motor control and lateral balance training may be beneficial. For overweight individuals, interventions prioritizing weight management and strength training to reduce mechanical load may be more appropriate. This research advances personalized fall risk assessment by demonstrating how BMI modifies the relationship between neural control markers and fall risk. Future fall prevention strategies should integrate neural control mechanisms with individual physiological characteristics to optimize intervention effectiveness and reduce fall-related morbidity in aging populations.

## Data Availability

The data analyzed in this study is subject to the following licenses/restrictions: the datasets used and/or analyzed are available from the corresponding author on reasonable request, due to privacy and consent restrictions. Requests to access these datasets should be directed to KK, kwkimmd@snu.ac.kr.
