# Supplementary material for: Neural control of anticipatory braking and lateral balance predicts fall risk in older adults: BMI-dependent mechanisms
Source: Front Aging Neurosci. 2026 Jan 12;17:1699584. doi: 10.3389/fnagi.2025.1699584 (PMC12832761; doi:10.3389/fnagi.2025.1699584)
Supplement: Supplementary file 1 [file Data_Sheet_1.docx]

Supplementary Material S1


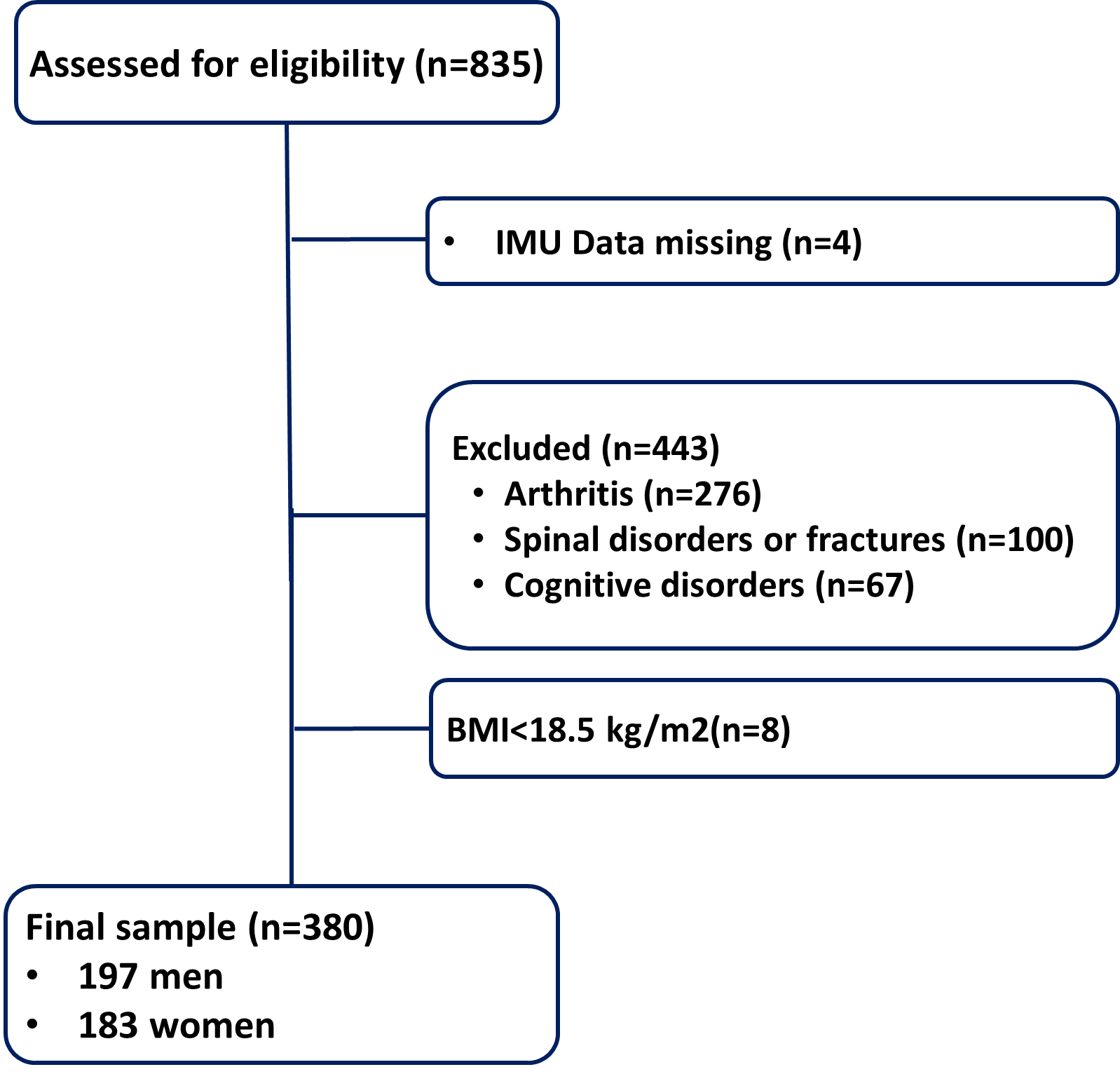


Supplementary Material


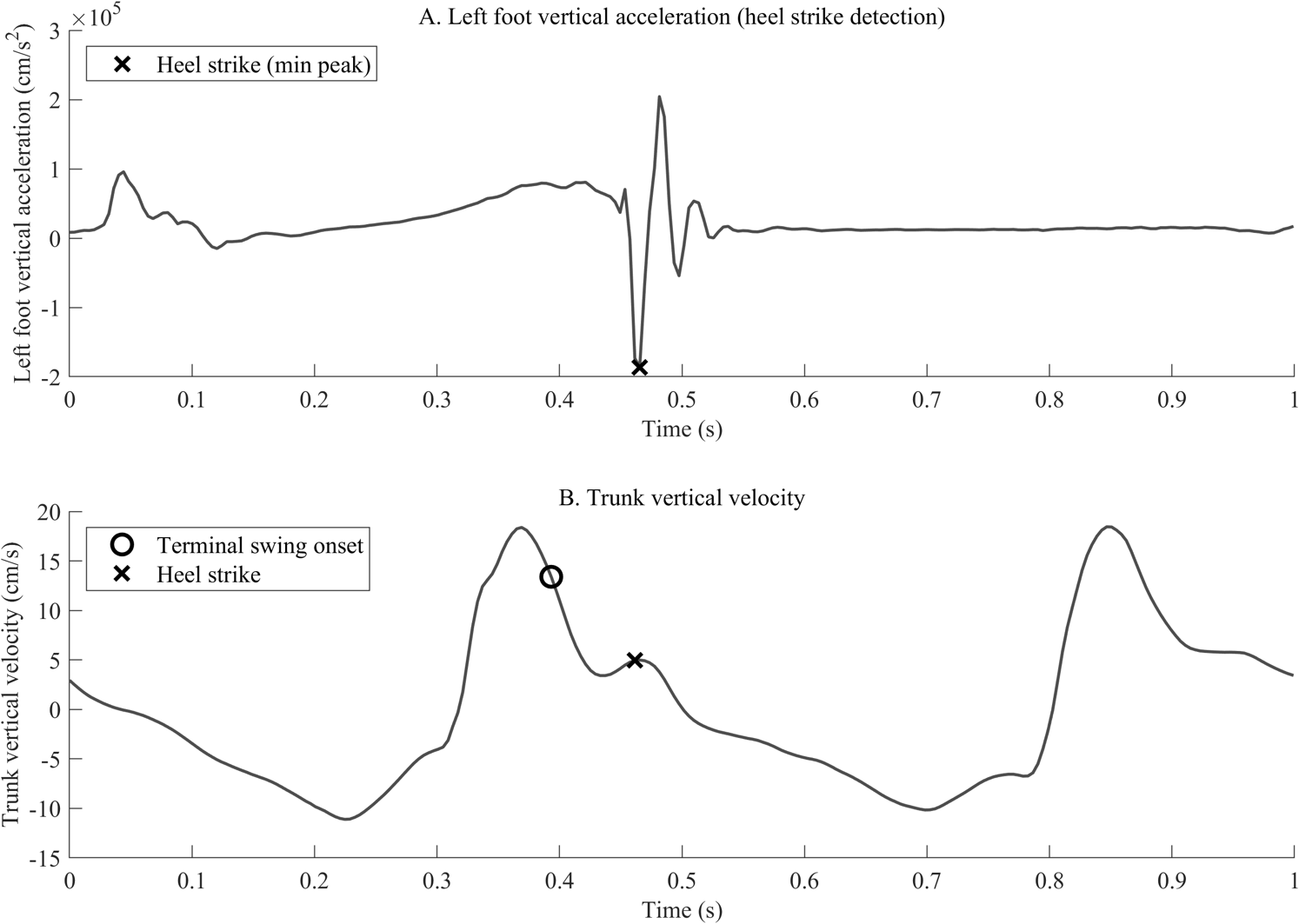


## ****Supplementary Figure S2. DCEL Scheme****

A illustrates the vertical acceleration signal of the left foot, where heel strike is identified as the minimum acceleration peak. B illustrates the trunk vertical velocity, marking the onset of terminal swing which is 13% before heel strike and the heel strike moment. These figures demonstrate how deceleration during terminal swing (DCEL) is derived from inertial measurement unit (IMU) data.


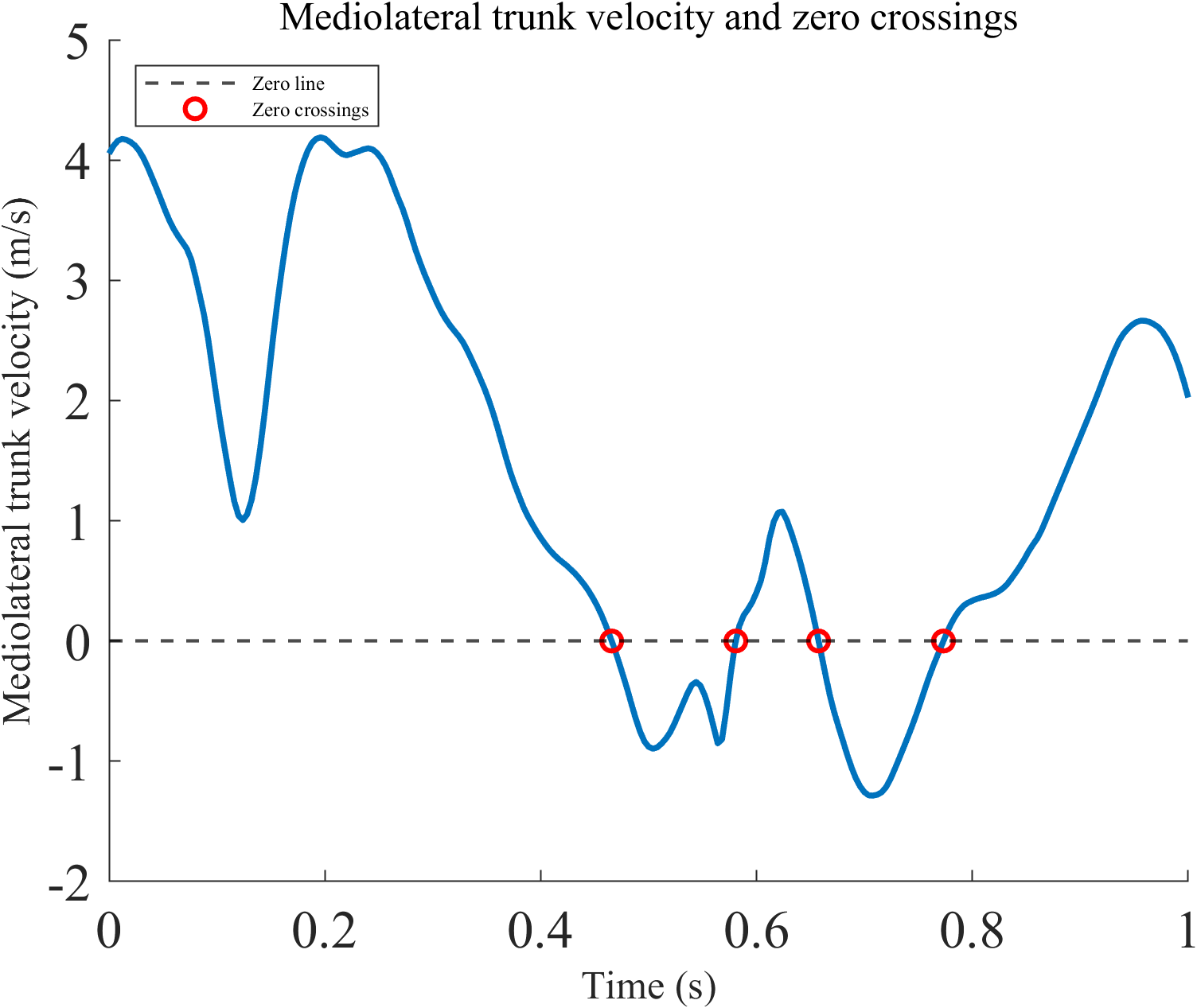


## ****Supplementary Figure S3. VZCC Scheme****

A short mediolateral trunk velocity segment is shown as an example to illustrate how zero-crossing events are detected. In the actual analysis, zero crossings are extracted from the continuous mediolateral velocity signal during steady-state walking and normalized by step length. The solid line represents mediolateral trunk velocity, and the dashed horizontal line indicates zero velocity. Red circles mark the zero crossing points, and the total number of crossings is defined as the velocity-based zero-crossing counts (VZCC), which reflects mediolateral corrective oscillations during gait.
