# Supplementary material for: Neural control of anticipatory braking and lateral balance predicts fall risk in older adults: BMI-dependent mechanisms
Source: Front Aging Neurosci. 2026 Jan 12;17:1699584. doi: 10.3389/fnagi.2025.1699584 (PMC12832761; doi:10.3389/fnagi.2025.1699584)
Supplement: Supplementary file 2 [file Table_1.doc]

STROBE Statement—Checklist of items that should be included in reports of ***cohort studies***

|  | Item No | Recommendation | Page  No. | Relevant text from manuscript |
| --- | --- | --- | --- | --- |
| **Title and abstract** | 1 | (*a*) Indicate the study’s design with a commonly used term in the title or the abstract | Page 2 | Line 26-29 |
|  |  | (*b*) Provide in the abstract an informative and balanced summary of what was done and what was found | Page 2 | Line 25-41 |
| Introduction | | |  |  |
| Background/rationale | 2 | Explain the scientific background and rationale for the investigation being reported | Page 3-5 | Line 49-110 |
| Objectives | 3 | State specific objectives, including any prespecified hypotheses | Page 6 | Line 124-129 |
| Methods | | |  |  |
| Study design | 4 | Present key elements of study design early in the paper | Page 6-7 | Line 141-148 |
| Setting | 5 | Describe the setting, locations, and relevant dates, including periods of recruitment, exposure, follow-up, and data collection | Page6-7 | Line 132-137, line 141-149 |
| Participants | 6 | (*a*) Give the eligibility criteria, and the sources and methods of selection of participants. Describe methods of follow-up | Page 6-7 | Methods - *Participants* |
|  |  | (*b*) For matched studies, give matching criteria and number of exposed and unexposed | NA | NA |
| Variables | 7 | Clearly define all outcomes, exposures, predictors, potential confounders, and effect modifiers. Give diagnostic criteria, if applicable | Page 7, 10 | Line 152-157; 184-189; 205-206; 219-221 |
| Data sources/ measurement | 8* | For each variable of interest, give sources of data and details of methods of assessment (measurement). Describe comparability of assessment methods if there is more than one group | Page 7-9 | Methods-*Gait Assessment and Data Processing and Analysis* |
| Bias | 9 | Describe any efforts to address potential sources of bias | Page 7 | Line 148-149 |
| Study size | 10 | Explain how the study size was arrived at | Page 6 | Line 140-141, Supplementary flow chart |
| Quantitative variables | 11 | Explain how quantitative variables were handled in the analyses. If applicable, describe which groupings were chosen and why | Page 9-11 | Methods – *Statistical Analysis* |
| Statistical methods | 12 | (*a*) Describe all statistical methods, including those used to control for confounding | Page 9-11 | Methods – *Statistical Analysis* |
|  |  | (*b*) Describe any methods used to examine subgroups and interactions | Page 9-11 | Methods – *Statistical Analysis* |
|  |  | (*c*) Explain how missing data were addressed | Page 11 | Line 241-244 |
|  |  | (*d*) If applicable, explain how loss to follow-up was addressed | Page 11 | Line 241-244 |
|  |  | (*e*) Describe any sensitivity analyses | Page 11 | Line 243-244 |
| Results | | |  |  |
| Participants | 13* | (a) Report numbers of individuals at each stage of study—eg numbers potentially eligible, examined for eligibility, confirmed eligible, included in the study, completing follow-up, and analysed | Page 11-12 | Line 251-253; 266-267 |
|  |  | (b) Give reasons for non-participation at each stage | Page 12 | Line 266-267, Supplementary figure S1 |
|  |  | (c) Consider use of a flow diagram | Page 12 | Line 266-267, Supplementary figure S1 |
| Descriptive data | 14* | (a) Give characteristics of study participants (eg demographic, clinical, social) and information on exposures and potential confounders | Page 11-12 | Line 251-267, Table 1 |
|  |  | (b) Indicate number of participants with missing data for each variable of interest | Page 11 | Line 241-242 |
|  |  | (c) Summarise follow-up time (eg, average and total amount) | Page 11 | Line 251-252 |
| Outcome data | 15* | Report numbers of outcome events or summary measures over time | Page 11 | Line 251-252 |
| Main results | 16 | (*a*) Give unadjusted estimates and, if applicable, confounder-adjusted estimates and their precision (eg, 95% confidence interval). Make clear which confounders were adjusted for and why they were included | Page 11-12 | Results – *Participant characteristics, Predictors of Fall Risk*, Table 2 |
|  |  | (*b*) Report category boundaries when continuous variables were categorized | Page 11 | Line 252-253 |
|  |  | (*c*) If relevant, consider translating estimates of relative risk into absolute risk for a meaningful time period | NA | NA |
| Other analyses | 17 | Report other analyses done—eg analyses of subgroups and interactions, and sensitivity analyses | Page 11-12 | Line 243-244, Results-*Predictors of Fall Risk*, *Stratified Analysis by BMI Group* |
| Discussion | | |  |  |
| Key results | 18 | Summarise key results with reference to study objectives | Page 13-14 | Discussion- 1^st^ -4^th^ paragraph |
| Limitations | 19 | Discuss limitations of the study, taking into account sources of potential bias or imprecision. Discuss both direction and magnitude of any potential bias | Page 19-20 | Line 432-456 |
| Interpretation | 20 | Give a cautious overall interpretation of results considering objectives, limitations, multiplicity of analyses, results from similar studies, and other relevant evidence | Page 14-18 | Line 322-328; 355-366; 402-420 |
| Generalisability | 21 | Discuss the generalisability (external validity) of the study results | Page 20 | Line 449-451; 466-471 |
| Other information | | |  |  |
| Funding | 22 | Give the source of funding and the role of the funders for the present study and, if applicable, for the original study on which the present article is based | Page 22 | Line 506-508 |

*Give information separately for exposed and unexposed groups.

**Note:** An Explanation and Elaboration article discusses each checklist item and gives methodological background and published examples of transparent reporting. The STROBE checklist is best used in conjunction with this article (freely available on the Web sites of PLoS Medicine at http://www.plosmedicine.org/, Annals of Internal Medicine at http://www.annals.org/, and Epidemiology at http://www.epidem.com/). Information on the STROBE Initiative is available at http://www.strobe-statement.org.
